# Prediction of successful weaning from renal replacement therapy in critically ill patients based on machine learning

**DOI:** 10.1080/0886022X.2024.2319329

**Published:** 2024-02-28

**Authors:** Qiqiang Liang, Xin Xu, Shuo Ding, Jin Wu, Man Huang

**Affiliations:** a General Intensive Care Unit, Second Affiliated Hospital of Zhejiang University School of Medicine, Hangzhou, PR China; b Key Laboratory of Multiple Organ Failure, China National Ministry of Education, Hangzhou, PR China

**Keywords:** RRT weaning, AKI, machine learning, multivariate logic regression, prediction model

## Abstract

**Background:**

Predicting the successful weaning of acute kidney injury (AKI) patients from renal replacement therapy (RRT) has emerged as a research focus, and we successfully built predictive models for RRT withdrawal in patients with severe AKI by machine learning.

**Methods:**

This retrospective single-center study utilized data from our general intensive care unit (ICU) Database, focusing on patients diagnosed with severe AKI who underwent RRT. We evaluated RRT weaning success based on patients being free of RRT in the subsequent week and their overall survival. Multiple logistic regression (MLR) and machine learning algorithms were adopted to construct the prediction models.

**Results:**

A total of 976 patients were included, with 349 patients successfully weaned off RRT. Longer RRT duration (7.0 *vs.* 9.6 d, *p* = 0.002, OR = 0.94), higher serum cystatin C levels (1.2 *vs.* 3.2 mg/L, *p* < 0.001, OR = 0.46), and the presence of septic shock (28.1% *vs.* 41.5%, *p* < 0.001, OR = 0.63) were associated with reduced likelihood of RRT weaning. Conversely, a positive furosemide stress test (FST) (60.2% *vs.* 40.7%, *p* < 0.001, OR = 2.75) and higher total urine volume 3 d before RRT withdrawal (755 *vs.* 125 mL/d, *p* < 0.001, OR = 2.12) were associated with an increased likelihood of successful weaning from RRT. Next, we demonstrated that machine learning models, especially Random Forest and XGBoost, achieving an AUROC of 0.95. The XGBoost model exhibited superior accuracy, yielding an AUROC of 0.849.

**Conclusion:**

High-risk factors for unsuccessful RRT weaning in severe AKI patients include prolonged RRT duration. Machine learning prediction models, when compared to models based on multivariate logistic regression using these indicators, offer distinct advantages in predictive accuracy.

## Background

Acute kidney injury (AKI) frequently afflicts critically ill patients in the intensive care unit (ICU). According to the latest epidemiological data, approximately 6–25% of AKI patients necessitate renal replacement therapy (RRT) due to the relentless progression of this condition [1]. Patients requiring RRT often experience extended ICU stays, heightened susceptibility to catheter-related infections, increased healthcare costs, and poorer clinical outcomes compared to their counterparts not requiring RRT. Restoring renal function becomes a pivotal consideration for critical care physicians managing AKI. However, the optimal timing for discontinuing RRT remains a subject of ongoing debate. The Kidney Disease: Improving Global Outcomes (KDIGO) guidelines, as of their 2012 Clinical AKI Practice Guide, underscored the importance of ceasing RRT when it is no longer necessary, either because intrinsic kidney function has recuperated sufficiently to meet the patient’s needs or because continued RRT aligns poorly with the patient’s care objectives [2]. Nevertheless, based on expert opinions, this guidance lacks precise directives regarding the timing of RRT weaning [2]. At present, there is a paucity of concrete laboratory markers to guide the process of RRT discontinuation, including indicators such as creatinine reduction or improved urine volume.

Kellum et al. delineated five AKI recovery models and defined recovery as the complete resolution of AKI by the time of hospital discharge or transfer to another department. Their analysis, based on a cohort of over 15,000 critically ill patients with AKI in stage KDIGO II/III [3], indicated that approximately 58.8% of AKI patients eventually recover during their hospitalization, albeit some cases of relapse [3]. This study underscores the potential for identifying variables predicting AKI recovery by analyzing clinical characteristics and constructing mathematical models. Consequently, numerous clinical investigations have constructed mathematical models to identify potential predictors of successful RRT weaning [4–6]. Commonly assessed laboratory variables in these studies include creatinine levels, endogenous creatinine clearance rate, 24-h urinary creatinine excretion, blood urea nitrogen (BUN), serum cystatin C, and N-terminal pro-brain natriuretic peptide. Simultaneously, various urine volume parameters are considered, encompassing total urine volume, 24-h urine output, urine output per hour following RRT cessation, and urine output without diuretics. Current research indicates that the most informative parameter is the 24-h urine volume preceding RRT discontinuation, with threshold values ranging from 500 to 2000 ml [4]. The heterogeneity in the criteria used by different studies in the literature for defining successful RRT weaning and the specific definitions of these variables should be highlighted, thereby diminishing the comparability between different investigations. However, important variables are relatively clear but not uniform, including urine volume and creatinine-related variables. These studies predominantly employed traditional multiple logistic regression (MLR) models, yielding areas under the receiver operating characteristic curve (AUROC) ranging from 0.78 to 0.87^4^. Thus, the overall predictive performance remains unsatisfactory.

Artificial intelligence (AI) and machine learning models, including but not limited to random forest (RF), gradient boosting, and deep neural networks, have attracted substantial attention because of their superior predictive capabilities. In contrast to traditional MLR models, AI models operate on novel logic paradigms, make more effective use of available data, and demonstrate enhanced predictive performance across diverse critical fields such as sepsis, acute respiratory distress syndrome, and AKI [7,8]. The 27th Acute Disease Quality Initiative consensus meeting, themed ‘Digital Health and Acute Kidney Injury (DHAKI)’, underscored the prominence of employing health information technology, AI, and related services in AKI prevention, diagnosis, recovery, and RRT management, signifying a current focal point in transformative research [9]. While ML models have been successfully applied in AKI for early diagnosis, mortality prediction, recovery assessment, and RRT timing, yielding accuracies ranging from 81% to 97%, there is a relative lack of ML models designed specifically for RRT weaning [10–13]. Leveraging our center’s extensive database and an established prediction model for severe AKI [14], we conducted data mining, including demographics, continuous renal replacement therapy (CRRT) during time, time to get on and off the CRRT, and laboratory indicators, vital signs, medication records before and after critical time nodes to develop a predictive model for RRT discontinuation in patients with severe AKI.

## Methods

Study design and setting: This study is a retrospective single-center investigation that utilized data from the Comprehensive ICU Database of the Second Affiliated Hospital of the Medical College of Zhejiang University (SHZJU-ICU). This large academic teaching hospital, located in southeast China, encompasses four districts in Hangzhou [14], boasting a total of 3800 beds. The General ICU spans across three of these districts, comprising independent wards with capacities of 26, 40, and 10 beds. Data from these three wards were pooled in 2021. The database aggregated information from approximately 18,000 critically ill patients, encompassing demographics, vital signs, clinical examinations, medication orders, clinical diagnoses, and medical records.

This study focused on patients diagnosed with severe acute renal injury who received RRT during hospitalization. Data collection was initiated upon the patient’s initiation of RRT, encompassing laboratory indicators and clinical status data recorded prior to RRT weaning. The success of RRT weaning was determined by the patient’s independence from RRT for 1 week following cessation and their survival during this period. Ethical approval for this study was granted by the Second Affiliated Hospital of Zhejiang University School of Medicine, with approval number IRB-2016-1511.

Study definition: The diagnosis of AKI adhered to KDIGO guidelines [2]. Successful weaning from RRT was defined as the continuous lack of requirement for RRT for 1 week, patient survival, and no utilization of regular hemodialysis within that week. Hemoperfusion, plasma exchange, blood lipid separation, bilirubin adsorption, and other specialized forms of RRT were not considered part of RRT in this study. Laboratory indices were assessed based on values recorded 1 week prior to RRT discontinuation, selecting the maximum or minimum values depending on clinical relevance. Medication history encompassed the 2 weeks leading up to RRT withdrawal, with continuous usage for at least 3 d. The definition of septic shock adhered to SEPSIS 2.0 criteria prior to 2018 and SEPSIS 3.0 criteria from 2018 onward [15,16]. A positive furosemide stress test (FST) was determined by a urine volume response following the administration of a high dose (>1 mg/kg) of furosemide within 1 week before RRT weaning [17]. A high vasoactive-inotropic score (VIS) denoted an accumulation of more than 30 points in vasoactive drug scores within 1 week before RRT discontinuation. The VIS score is a widely accepted standardized quantitative measure of vasoactive drug usage [18]. Invasive catheterization is defined as any procedure that involves accessing the blood vessels, including central venous catheter placement, RRT catheter insertion, all angiographic procedures, and interventional therapies. Invasive procedures refer to interventional techniques that do not invade blood vessels, such as endoscopic interventions, lumbar puncture and catheter placement, and ultrasound-guided percutaneous interventions.

Model construction and validation: Data collection and management were executed using MySQL and Navicat database tools, while data processing and modeling were carried out using R (version 3.5.3, St. Louis, MO) and R Studio (version 1.2.1335, Boston, MA) with packages including ‘mass’, ‘vcd’, ‘rpart’, and ‘rpart.plot’ [19]. Multivariable logistic regression and three machine learning algorithms, decision tree (DT), support vector machine (SVM), RF, and XGBoost, were selected to establish the models [8,20]. Relevant variables were extracted from the database using Navicat software and organized and processed through R software. This entailed variable screening and both simple and multiple imputations. Variables with missing values exceeding 40% were excluded, and cases with missing variables exceeding 50% were removed. Variables with missing values ranging from 10% to 40% were addressed through multiple imputations, while those with less than 10% missing data were filled *via* simple interpolation [21,22]. All data are randomly split according to proportion, with 70% of the data training, 15% for validation, and 15% for testing. Model construction encompassed the utilization of MLR, DT, SVM, RF, and extreme gradient boosting (XGBoost) algorithms. The four machine learning algorithms we choose are the common supervised learning algorithms, DT, SVM, RF, and XGBoost, respectively. The DT model produces different branches by calculating the characteristics of independent variables and divides the data into multiple subsets with similar characteristics to achieve classification [8]. RF is a comprehensive decision of hundreds of DTs, and each DT is independent of each other. The accuracy is higher than the DT. XGBoost is a set algorithm of numerous DTs but is an improved version of the Gradient Boosting algorithm with highly efficient, flexible. Different from RFs, these DTs are interrelated, and it is also very abstract. Five hundred trees were constructed and the exhaustive method was used to adjust the parameters in the RF. Model parameter tuning and dataset validation were carried out *via* five-fold cross-validation, with model performance evaluated using the test set spanning the past 2 years [20].

Statistical analysis: Population characteristics were reported as medians and interquartile ranges (IQR) for skewed data and as means and standard deviations (SDs) for normally distributed data. Independent sample t-tests were employed for normally distributed data, while the Wilcoxon rank-sum test was utilized for non-normally distributed data. Dichotomous variables were assessed using the χ2 test, with statistical significance defined as a *p* value less than 0.05. Non-normally distributed data underwent exponential and logarithmic transformations. Model performance was evaluated using parameters such as the AUROC, accuracy, sensitivity, specificity, and F1-score.

## Results

**Figure 1.**
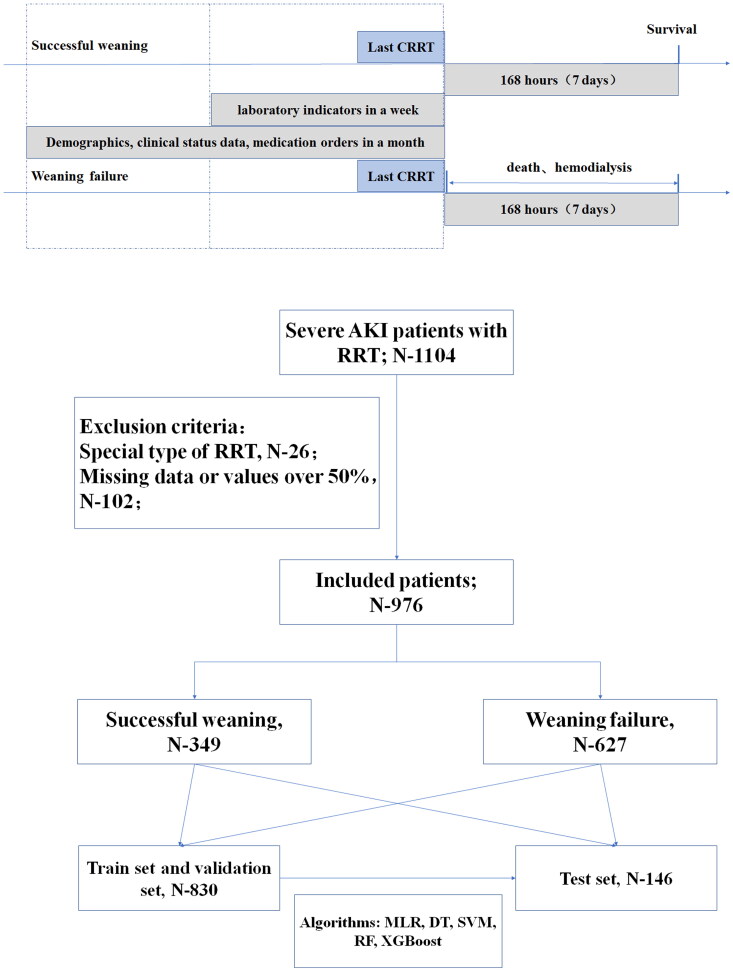
The research flow chart and schematic diagram of sampling time range and outcome of RRT withdrawal with variables.

A total of 1104 patients diagnosed with severe AKI received RRT during their hospitalization, with 26 patients undergoing specialized forms of RRT and 102 patients with substantial missing data excluded. Ultimately, 976 patients were included in the study, comprising patients who successfully weaned off RRT (*n* = 349) or did not achieve RRT withdrawal (*n* = 627). Detailed information is illustrated in Figure 1.

No significant differences were observed in gender, age, or body mass index (BMI) between the two groups. However, there is a lower proportion of cardio-cerebrovascular accidents in the successful weaning group (2.3% *vs.* 8.0%, *p* < 0.001; 17.8% *vs.* 23.4%, *p* = 0.038, respectively). Interestingly, patients who underwent surgical procedures were more likely to successfully wean off RRT (21.2% *vs.* 11.8%, *p* < 0.001). While the length of hospital stay did not differ, patients who achieved successful RRT withdrawal exhibited a higher 30-d survival rate for all causes (40.4% *vs.* 30.6%, *p* = 0.002). Univariate analysis revealed statistically significant clinical indicators, including maximum creatinine, maximum urea nitrogen, minimum hemoglobin, maximum C-reactive protein, total bilirubin, serum cystatin C, total protein, fibrinogen, urine volume within the 72 h preceding RRT weaning, RRT duration, positive FST results, aspirin history, malignant tumors, septic shock, high VIS, and history of invasive procedures. Additional details are provided in Table 1.

**Table 1.** Baseline characteristic and variables of weaning from RRT in severe acute kidney injury patients in ICU.

Variables	Successful weaning from RRT; *N* = 349	Failure of RRT weaning; *N* = 627	*p*	OR [95%CI]
Gender, male	226 (64.8%)	417 (66.5%)	0.580	0.972 [0.88–1.08]
Age (years)	62.0 ± 17.6	61.2 ± 17.3	0.514	
BMI (kg/m^2^)	22.6 ± 3.8	22.5 ± 3.9	0.43	
Primary disease				
Infections	111 (31.8%)	192 (30.6%)	0.70	1.06 [0.79–1.40]
Trauma	44 (12.6%)	85 (13.6%)	0.67	0.92 [0.62–1.36]
Cardio-cerebrovascular accident	8 (2.3%)	50 (8.0%)	<0.001	0.27 [.012–0.58]
Heart disease	62 (17.8%)	147 (23.4%)	0.038	0.71 [0.51–0.98]
Internal medicine diseases	50 (14.3%)	79 (12.6%)	0.45	1.16 [0.79–1.70]
Postoperative diseases	74 (21.2%)	74 (11.8%)	<0.001	2.01 [1.41–2.86]
Diabetes mellitus	62 (17.7%)	109 (17.4%)	0.881	1.02 [0.73–1.45]
Malignant tumor	56 (16.0%)	69 (11.0%)	0.024	1.54 [1.06–2.26]
Hypertension	140 (40.1%)	269 (42.9%)	0.397	0.89 [0.68–1.16]
Scr_max_ (μmol/L, IQR)	169.0 [114.0–296.0]	226.0 [212.0–321.0]	<0.001	
Scr_min_ (μmol/L, IQR)	84.0 [53.0–131.0]	82.0 [54.0–128.0]	0.926	
BUN_max_ (mmol/L, IQR)	14.8 [9.3–24.1]	21.8 [14.2–33.0]	<0.001	
WBC_max_ (10^9/L, IQR)	14.2 [9.4–22.2]	15.6 [10.3–22.8]	0.30	
Hb_min_ (g/L, IQR)	65.0 [56.0–78.0]	63.0 [52.0–75.0]	0.003	
PLT_min_ (10^9/L, IQR)	42.0 [14.0–111.0]	48.0 [18.0–97.0]	0.996	
LYM_max_ (10^9/L, IQR)	0.9[0.4–1.0]	0.9 [0.3–1.0]	0.115	
CRP_max_ (mg/L, IQR)	73.8 [32.5–143.0]	109.4 [48.6–182.4]	<0.001	
ALT_max_ (U/L, IQR)	50.0 [24.0–138.0]	50.0 [24.0–202.0]	0.659	
AST_max_ (U/L, IQR)	67.0 [37.0–264.0]	91.0 [40.0–356.0]	0.549	
Serum cystatin C_max_ (mg/L, IQR)	1.2 [1.0–2.2]	3.2 [1.7–4.3]	<0.001	
Tbil_max_ (μmol/L, IQR)	23.0 [16.0–64.0]	27.1 [16.8–49.4]	0.035	
Total protein_max_ (μmol/L, IQR)	48 [42.6–55.4]	50.0 [43.9–56.4]	0.003	
Albumin_max_ (g/L, IQR)	25.0 [21.7–28.4]	25.8 [22.0–29.4]	0.007	
PT_max_ (s, IQR)	20.6 [15.8–26.1]	21.6 [16.9–28.8]	<0.001	
APTT_max_ (s, IQR)	76.1 [59.1–106.1]	76.0 [57.6–105.3]	0.795	
FIG_max_ (g/L, IQR)	2.1 [1.3-3.7]	2.8 [1.6-4.2]	<0.001	
D-dimer_max_ (ug/L, IQR)	10390.0 [4500.0–20000.0]	10720.0 [5390.0–20000.0]	0.755	
Urine volume in 72 h before weaning from RRT (mL/d, IQR)	755.0 [105.0–2350.0]	125 [20.0–300.0]	<0.001	
CRRT duration (d, IQR)	7.0 [2.0–15.0]	9.6 [2.5–16.8]	<0.001	
Positive FST	210 (60.2%)	255 (40.7%)	<0.001	2.20 [1.69–2.88]
History of glucocorticoid	129 (36.9%)	213 (34.0%)	0.348	1.14 [0.86–1.50]
History of aspirin	42 (12.0%)	113 (18.0%)	0.014	0.62 [0.42–0.91]
Septic shock	98 (28.1%)	260 (41.5%)	<0.001	0.55 [0.42–0.73]
High VIS	157 (45.0%)	350 (55.8%)	0.001	0.65 [0.49–0.84]
Invasive catheterization	203 (58.2%)	366 (58.4%)	0.95	0.99 [0.76–1.29]
History of ECMO	25 (7.2%)	51 (8.1%)	0.588	0.87 [0.53–1.43]
Invasive procedure	128 (36.7%)	349 (55.7%)	<0.001	0.46 [0.35–0.60]
Operation history	157 (45.0%)	272 (43.4%)	0.628	1.07 [0.82–1.39]
Ward admission	86 (24.6%)	111 (17.7%)	0.01	1.52 [1.11–2.09]
Emergency admission	269 (77.1%)	523 (83.4%)	0.015	0.67 [0.48–0.93]
Survival rate in 30 d	141 (40.4%)	192(30.6%)	0.002	1.53 [1.17–2.02]
Hospitalization frequency (d, IQR)	2.0 [1.0–4.0]	2.2 [1.0–5.0]	0.088	
LOS (d, IQR)	13.9 [5.7–28.0]	17.0 [4.5–32.0]	0.519	

RRT: renal replacement therapy; CRRT: continuous renal replacement therapy; BMI: body mass index; Scr: serum creatinine; BUN: blood urea nitrogen; WBC: white blood cell count; Hb: hemoglobin; PLT: platelet; LYM: lymphocyte count; CRP: C-reactive protein; ALT: glutamic-pyruvic transaminase; AST: glutamic oxalacetic transaminase; Tbil: total bilirubin; PT: prothrombin time; APTT: activated partial thromboplastin time; FIG: fibrinogen; FST: furosemide stress test; VIS: vasoactive-inotropic score; ECMO: Extracorporeal Membrane Oxygenation; LOS: length of stay

The correlation between these variables was generally low, as depicted in Supplemental Figure. Multivariate analysis demonstrated that longer RRT duration (7.0 *vs.* 9.6 d, *p* = 0.002, OR = 0.94, 95%CI: 0.92–0.96), higher C-reactive protein levels (73.8 *vs.* 109.4 mg/L, *p* = 0.003, OR = 0.91, 95%CI: 0.90–0.92), elevated serum cystatin C (1.2 *vs.* 3.2 mg/L, *p* < 0.001, OR = 0.46, 95%CI: 0.40–0.53), septic shock (28.1% *vs.* 41.5%, *p* < 0.001, OR = 0.63, 95%CI: 0.45–0.89), and a history of invasive procedures (36.7% *vs.* 55.7%, *p* < 0.001, OR = 0.33, 95%CI: 0.25–0.46) were associated with a reduced likelihood of successful RRT weaning. Conversely, positive FST results (60.2% *vs.* 40.7%, *p* < 0.001, OR = 2.75, 95%CI: 1.98–3.83) and higher total urine volume 3 d before RRT withdrawal (755 *vs.* 125 mL/d, *p* < 0.001, OR = 2.12, 95%CI: 1.03–3.34) were associated with an increased likelihood of successful RRT weaning. Further details are provided in Table 2.

**Table 2.** Parameters in the multivariable logistic regression model of weaning from RRT in severe AKI patients.

Value	*p*	OR [95%CI]
CRRT duration	0.002	0.94 [0.92–0.96]
CRP_max_	0.003	0.91 [0.90–0.92]
Homocysteine_max_	<0.001	0.46 [0.40–0.53]
Urine volume in 72 h before weaning from RRT	<0.001	2.12 [1.03–3.34]
Pt_max_	<0.001	0.97 [0.96–0.98]
Positive FST	<0.001	2.75 [1.98–3.83]
Septic shock	<0.001	0.63 [0.45–0.89]
Invasive procedure	<0.001	0.33 [0.23–0.46]

RRT: renal replacement therapy; CRRT: continuous renal replacement therapy; PT: prothrombin time; CRP: C-reactive protein; FST: furosemide stress test; OR: odds ratio; CI: confidence interval

**Figure 2.**
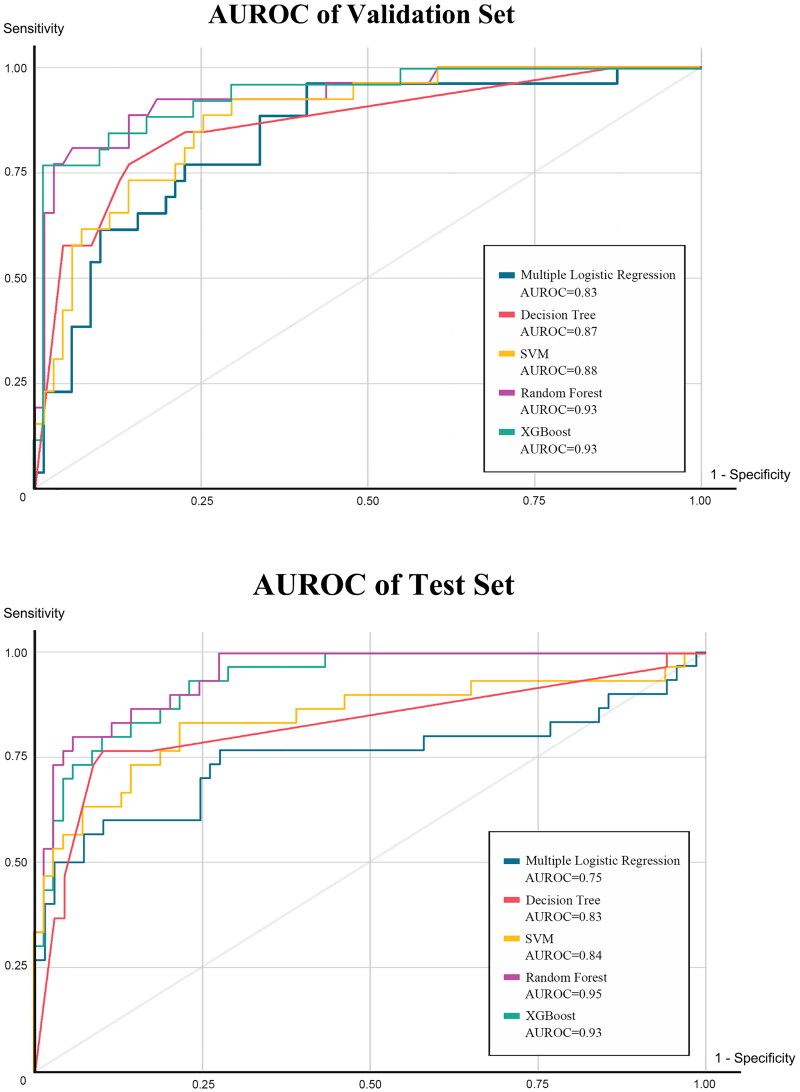
The AUROC curve of validation set and test set in prediction model of weaning from RRT.

**Figure 3.**
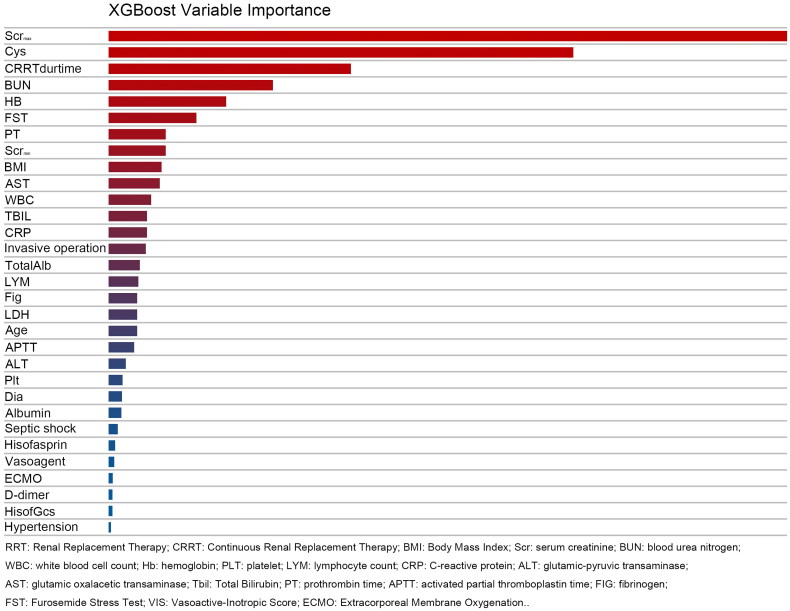
Visual histogram of variable importance of XGBoost.

Among the four machine learning prediction models and the multivariate logistic regression (MLR) model, the AUROC of the MLR model was 0.83 in the validation set and 0.75 in the training set. In comparison, the machine learning models performed notably better, especially the RF model with AUROCs of 0.93 in the validation set and 0.95 in the test set. The XGBoost model also demonstrated superior performance, with AUROCs of 0.93 in the validation and test set. Accuracy was consistently higher in the machine learning models compared to the MLR model, with the RF model achieving an impressive accuracy of0.889, significantly outperforming the latter (0.818). The RF model demonstrated excellent specificity, reaching 0.928. Detailed results are presented in Table 3. The AUROC curve for the model is depicted in Figure 2 and the variable importance for XGBoost is illustrated in Figure 3.

**Table 3.** Model validation results with machine learning algorithms.

Validation	AUROC	Accuracy	Sensibility	Specificity	PPV	NPV	F1
MLR	0.83	0.814	0.615	0.887	0.667	0.863	0.640
DT	0.87	0.835	0.731	0.873	0.679	0.899	0.704
SVM	0.88	0.825	0.654	0.887	0.680	0.875	0.667
RF	0.93	0.907	0.808	0.944	0.840	0.931	0.824
XGBoost	0.93	0.887	0.769	0.930	0.800	0.917	0.784
Test							
MLR	0.75	0.818	0.567	0.928	0.773	0.831	0.654
DT	0.83	0.859	0.767	0.899	0.767	0.899	0.767
SVM	0.84	0.808	0.633	0.884	0.704	0.847	0.667
RF	0.95	0.889	0.800	0.928	0.828	0.914	0.814
XGBoost	0.93	0.869	0.800	0.899	0.774	0.912	0.787

## Discussion

In this study, we focused on patients with severe AKI who underwent RRT. We employed traditional MLR and machine learning prediction models to assess the likelihood of successful weaning off RRT, defined as being free of RRT for over 1 week after the last RRT session while surviving. This definition is in agreement with previous cohort studies [4,23–26], which typically adopted varying timeframes for weaning, ranging from 2 to 30 d. We selected the commonly used and relatively versatile definition of 1 week, coupled with the survival requirement, based on the size and quality of prior research.

Most retrospective studies examining RRT weaning have relied on the traditional MLR model. While these studies encompassed a wide range of variables, the primary focus has been evaluating the relative importance of different factors [4–6]. Kidney-related variables include various forms of creatinine, endogenous creatinine clearance, 24-h urinary creatinine, BUN, urine volume in different periods, etc. Renal biomarkers with high frequency include Serum cystatin C, NT-proBNP, IL-8, and IL-6, selected variables in these studies [4,23–26]. The variables selected by the MLR model in our study included serum cystatin C, urine volume in 72 h before weaning from RRT, and positive FST, similar to those in previous studies. While the maximum creatinine value in the past week showed statistical significance during univariate analysis, it was optimized during the model construction, and potential collinearity with other variables could not be ruled out. Of interest, among the important variables identified by XGBoost, the maximum value of creatinine remained one of the most influential, followed by serum cystatin C. The XGBoost model, based on DTs, is often considered a ‘black box’, which has been a major criticism of machine learning [11].

Consistent with the literature, our study provided compelling evidence that machine learning prediction models consistently outperform the MLR model in predicting RRT weaning, validating the trend observed in prior research. In this respect, Xiujuan Zhao and her colleagues employed a dataset comprising 12,321 AKI patients from the Medical Information Mart for Intensive Care (MIMIC)-IV database. They defined renal function recovery within 72 h after AKI onset as short-term reversibility and documented a recovery rate of 67.9% [27]. The maximum creatinine value within 24 h emerged as a common independent predictor of renal function recovery and short-term reversibility of AKI. Compared to the traditional MLR model, the RF model exhibited superior performance, with AUROCs of 0.8295 and 0.7683 for predicting renal function recovery and early recovery, respectively [27]. During our analysis of AKI data within the MIMIC database, the proportion of patients with AKI KDIGO stage I was considerably higher than those with AKI KDIGO stage II/III^14^. However, patients with AKI stage I often did not require RRT and can experience self-reversal of their condition, limiting the clinical significance of predicting RRT weaning in this group. Justin Y Lu and other scholars conducted a clinical analysis of 3296 COVID-19 patients with AKI and stratified analysis according to the recovery time of AKI. It was found that patients had a higher risk of adverse cardiovascular and renal outcomes with slow recovery of AKI. In this study, 70% of AKI patients recovered within 7 d, which is gratifying, suggesting that most mild AKI can recover. However, the RRT rate of patients who recovered within 7 d in this study was less than 2%, while the rate would raise to 43.8% of patients with delayed recovery [28]. Patients with severe AKI have high utilization rate of CRRT, high mortality rate, and high clinical cost, which are the key targets to be overcome. Consequently, our study primarily focused on AKI KDIGO stage II/III patients, as they were more likely to necessitate RRT, making predictions regarding RRT weaning particularly relevant for clinicians. This approach is in agreement with that adopted by Pattharanitima et al., who analyzed the MIMIC database, revealing that 205 cases (30%) out of 684 AKI patients treated with RRT successfully weaned from RRT. They defined successful weaning as the absence of RRT usage and survival for a week before discharge [29]. Their study employed seven modeling algorithms, including MLR, Random Trees (RT), SVM, XGBoost, Adaptive Boosting (AdaBoost), Multilayer Perceptron (MLP), and MLP with Long Short-Term Memory (MLP + LSTM). The highest AUROC was achieved by MLP + LSTM at 0.70 (95% CI: 0.67–0.73), while LR yielded with an AUROC of only 0.57 (0.52–0.62) [29]. It should be noted that, in the MIMIC database, the proportion of patients receiving RRT was relatively low (1.7%). This scarcity of data can potentially lead to models with limited predictive abilities, which may not be suitable for direct clinical application [29]. To establish our prediction model for severe AKI, we leveraged various machine learning models, among which XGBoost exhibited a superior AUROC of 0.86 compared to the MLR model [14]. Furthermore, Kang et al. employed the RF model to predict the mortality of patients receiving RRT, achieving an AUROC curve of 0.784. Their model outperformed previous scoring models, including the Acute Physiological and Chronic Health (APACHE II) scores and Sequential Organ Failure Assessment (SOFA) scores [30]. Hsin-Hsiung Chang and Pei-Shan Hung had come to similar conclusions on the mortality of CRRT patients by comparing traditional scoring and machine learning algorithms [31,32].

The application of AI in AKI research, encompassing predictions related to AKI occurrence, prognosis, renal function recovery, survival without RRT, 6-h hypotension time during RRT, and more, has consistently outperformed traditional models [11]. Based on the data from two centers, Justin Y Lu found the AI algorithm has excellent diagnostic efficiency in predicting the occurrence of AKI in patients with COVID-19 [33]. Current supervised learning models predominantly focus on classification tasks, such as distinguishing between survival and death or the occurrence and nonoccurrence of events. The strength of AI models lies in their ability to comprehensively incorporate a wide array of variables without oversimplification, relying on robust computational capabilities and extensive datasets. This approach allows them to extract more information and achieve greater accuracy in classification tasks [11,20]. In contrast, the MLR model tends to emphasize a narrower selection of variables to optimize model fit, and its performance hinges on choosing these variables, often leading to the exclusion of meaningful but less prominent variables. While simplifying variables may enhance operational efficiency and conciseness, it entails the loss of valuable information.

Notably, there is a paucity of research focusing on RRT weaning using machine learning, with more attention directed toward predicting AKI morbidity and mortality [34]. Although not being CRRT withdrawal often means clinical death, and there is a high probability that survival can be weaning from CRRT, there is still a difference in the meaning of survival time. Our research extends the application of machine learning prediction model in AKI and is an important part of AKI intelligent management. One of the key challenges in our model and other published AI prediction models is the absence of external validation. It has been mentioned in the discussion, but the main defect of the current research. We plan to optimize our severe AKI prediction model through prospective studies to address this gap. Additionally, our reliance on retrospective data poses a limitation, as the dataset may not encompass newer biomarkers of AKI introduced in recent years due to data availability constraints. We have a small amount of data for machine learning, which may affect the prediction effect of the model, and our data type, mainly laboratory indicators, is relatively simple, and other relatively difficult to collect but more important indicators, including urine volume, hourly input and output, may be deleted because of missing data. In addition, our algorithm does not make use of the large but messy content of continuous variables and text variables, and it may also lead to a decline in accuracy.

## Conclusion

High-risk factors for failure in weaning from RRT in severe AKI patients include long duration of RRT, septic shock, low urine volume in 72 h before weaning from RRT, and negative FST. Compared with the MLR model based on these indicators, the machine learning-based prediction model yielded more advantages in terms of sensitivity and specificity.

## Supplementary Material

Supplemental Material

## Data Availability

The datasets used and/or analyzed during this study are available from the corresponding author on reasonable request.
