# Supplementary figures and images for: Prediction of successful weaning from renal replacement therapy in critically ill patients based on machine learning
Source: Ren Fail. 2024 Feb 28;46(1):2319329. doi: 10.1080/0886022X.2024.2319329 (PMC10903749; doi:10.1080/0886022X.2024.2319329)

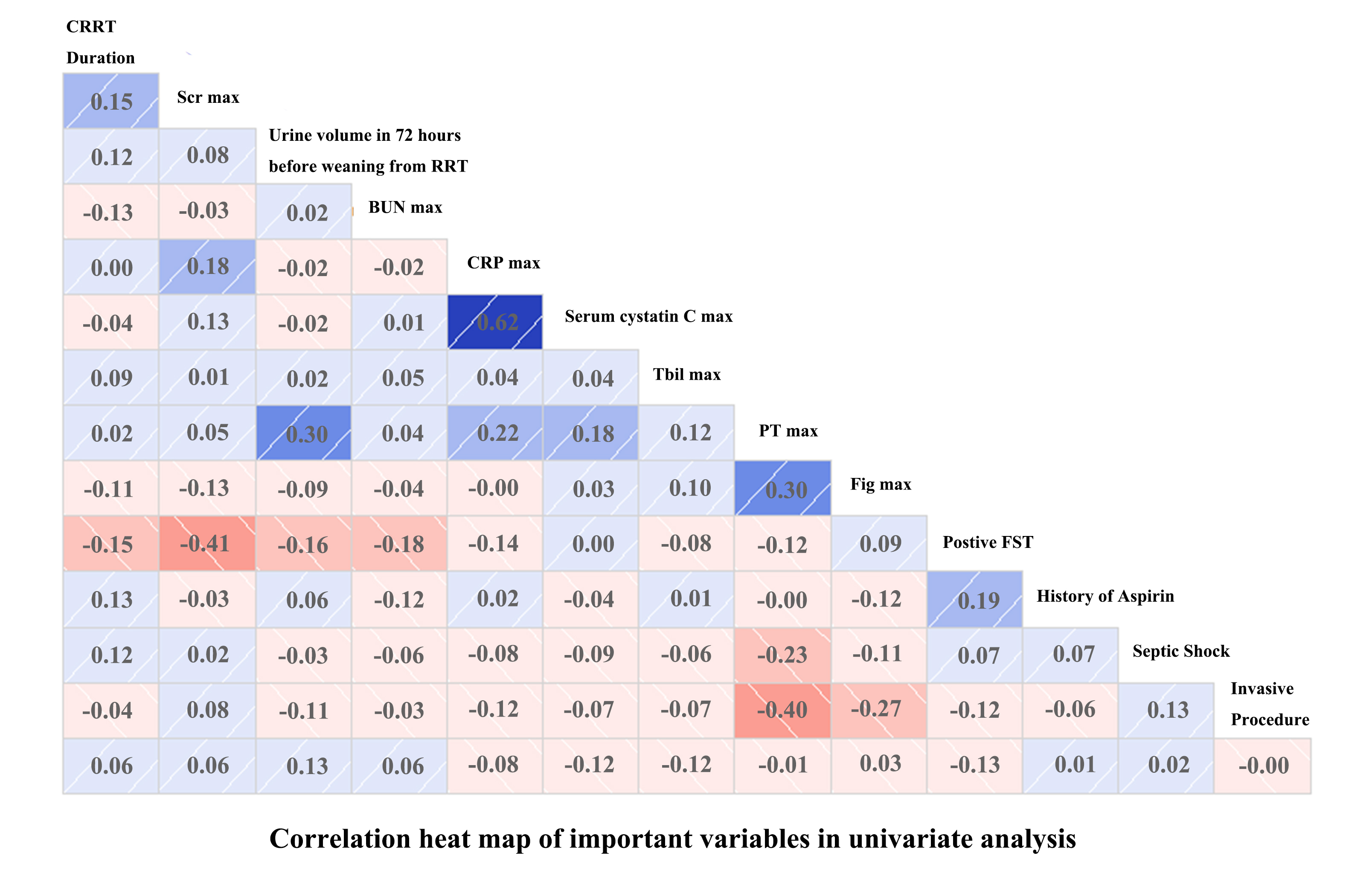

Supplement: Supplemental Material [file IRNF_A_2319329_SM9977.jpg]
